# Identification and characterization of the three homeologues of a new *sucrose transporter* in hexaploid wheat (*Triticum aestivum* L.)

**DOI:** 10.1186/1471-2229-13-181

**Published:** 2013-11-16

**Authors:** Kirandeep K Deol, Shalini Mukherjee, Feng Gao, Anita Brûlé-Babel, Claudio Stasolla, Belay T Ayele

**Affiliations:** 1 Department of Plant Science, 222 Agriculture Building, University of Manitoba, Winnipeg, Manitoba R3T 2 N2, Canada

**Keywords:** Homeologous genes, *TaSUT2*, Sucrose transporter, Gene expression, Source tissues, Developing seeds, Wheat

## Abstract

**Background:**

Sucrose transporters (SUTs) play important roles in regulating the translocation of assimilates from source to sink tissues. Identification and characterization of new *SUT*s in economically important crops such as wheat provide insights into their role in determining seed yield. To date, however, only one *SUT* of wheat has been reported and functionally characterized. The present study reports the isolation and characterization of a new *SUT*, designated as *TaSUT2*, and its homeologues (*TaSUT2A*, *TaSUT2B* and *TaSUT2D*) in hexaploid wheat (*Triticum aestivum* L.).

**Results:**

*TaSUT2A* and *TaSUT2B* genes each encode a protein with 506 amino acids, whereas *TaSUT2D* encodes a protein of 508 amino acids. The molecular mass of these proteins is predicted to be ~ 54 kDA. Topological analysis of the amino acid sequences of the three homeologues revealed that they contain 12 transmembrane spanning helices, which are described as distinct characteristic features of glycoside-pentoside-hexuronide cation symporter family that includes all known plant SUTs, and a histidine residue that appears to be localized at and associated conformationally with the sucrose binding site. Yeast SUSY7/*ura3* strain cells transformed with *TaSUT2A*, *TaSUT2B* and *TaSUT2D* were able to uptake sucrose and grow on a medium containing sucrose as a sole source of carbon; however, our subcellular localization study with plant cells revealed that TaSUT2 is localized to the tonoplast. The expression of *TaSUT2* was detected in the source, including flag leaf blade, flag leaf sheath, peduncle, glumes, palea and lemma, and sink (seed) tissues. The relative contributions of the three genomes of wheat to the total expression of *TaSUT2* appear to differ with tissues and developmental stages. At the cellular level, *TaSUT2* is expressed mainly in the vein of developing seeds and subepidermal mesophyll cells of the leaf blade.

**Conclusion:**

This study demonstrated that TaSUT2 is a new wheat SUT protein. Given that TaSUT2 is localized to the tonoplast and sucrose is temporarily stored in the vacuoles of both source and sink tissues, our data imply that TaSUT2 is involved in the intracellular partitioning of sucrose, particularly between the vacuole and cytoplasm.

## Background

Sucrose is the major transported form of sugar in plants. The deposition of starch, which is the main determinant of yield in cereal crops, in seeds is dependent on the supply of this sugar molecule from source tissues through the phloem. The phloem consists of sieve elements (SE) and companion cells (CC) connected by numerous intercellular connections called plasmodesmata and form a SE-CC complex [1]. The SE is responsible for long distance transport of sucrose and other organic materials, whereas the CCs supply proteins and energy to the SEs [2,3]. Phloem loading and unloading of sucrose takes place symplastically and apoplastically. The symplastic transport involves cell to cell movement of sucrose via plasmodesmatal connections, while the apoplastic transport involves active sucrose movement across membranes via sucrose transporter (SUT) proteins [3,4], and mainly takes place in the absence of plasmodesmatal connections. Genes encoding SUTs have been identified from a number of plant species including cereal crops such as rice (*Oryza sativa*) [5], barley (*Hordeum vulgare*) [6], maize (*Zea mays*) [7] and wheat (*Triticum aestivum*) [8], and form small gene families [9]. All the plant SUTs identified to date are members of the glycoside-pentoside-hexuronide (GPH) cation symporter family, which is part of the major facilitator superfamily characterized by 12 transmembrane spanning helices [10,11]. Furthermore, the first extracellular loop of plant SUTs contains a histidine residue, such as His-65 of the Arabidopsis SUT1 that serves as a target for diethyl pyrocarbonate (DEPC) mediated inhibition of sucrose transport activity [12]. It has been shown that this residue is substrate protected from the inhibition reaction induced by DEPC. This along with the ability of eliminating SUTs’ sensitivity to DEPC by substituting histidine with other amino acids without affecting the transport activity of SUTs indicate that the DEPC sensitive histidine residue is localized at or associated conformationally with the sucrose binding site.

Spatiotemporal expression analyses of the *SUT*s in cereal crops have provided insights into their physiological roles during seed development. For example, *SUT1* of maize is highly expressed in leaf blades, leaf sheaths, culms, and husks and pedicels of the ear during reproductive growth of maize; and the expression of *ZmSUT1* in the leaf blades was shown to increase as the level of photoassimilates increases, reflecting its role in phloem loading [7,13]. Consistent with this observation, impaired phloem loading in the *zmsut1* mutant causes accumulation of carbohydrates in the leaf that led to chlorosis, senescence and reduced plant growth [14]. The three *TaSUT1* homeologues, which reside on chromosome 4 of the wheat A, B and D genomes, are found to be expressed at similar levels in flag leaf blades, leaf sheaths and internodes [8], suggesting their equal roles in phloem loading of sucrose. The transcript abundance of *TaSUT1* in these tissues was highest before heading and decreased immediately after flowering, when an increased level of its transcript was evident in the developing seeds. A study with symplastic fluorescein tracer has shown the lack of plasmodesmatal connections in the SE-CC complex of wheat flag leaf [15], supporting the hypothesis that *TaSUT1* is involved in phloem loading of sucrose. In addition, all *SUT*s of rice [5,16] and the two known *SUT* of barley [6] are shown to be expressed in source leaves. Previous studies have also shown that non-foliar tissues of wheat florets, including the glume, lemma and palea, possess photosynthetic ability and contribute 10% to 44% of photoassimilates destined to wheat seeds [17,18]. In agreement with this, transcripts of *TaSUT1* were detected in the glumes of wheat ear both before and after heading, although at lower levels than that observed in the flag leaf blades and sheaths [8], suggesting its role in phloem loading of the sucrose produced in the spike.

Leaf sheaths of cereal crops connect leaf blades to the stem nodes, thereby forming phloem conduits that serve as pathways for long distance transport of photoassimilates [15]. Along with internodes, they also act as a temporary storage of excess carbohydrates produced during the early stages of seed filling in the form of water-soluble carbohydrates, mainly fructans. Approximately 50% of the photoassimilates destined to seed filling in wheat appears to be temporarily stored in the leaf sheaths and internodes prior to remobilization during active seed filling, when photoassimilate supply from the leaf is not enough to meet the sink demand [19]. A role for reloading sucrose into the phloem during the remobilization process has been suggested for *OsSUT1* and *TaSUT1* as their transcripts are present in the leaf sheath and stem tissues of rice and wheat, respectively [5,15,16]. Given that carboxyfluorescein dye moves symplastically out of the phloem in wheat internode [15], the presence of *TaSUT1* transcripts in this tissue also suggests its role in retrieving sucrose leaked out into the phloem apoplasm, and thereby providing an efficient photoassimilate translocation mechanism.

Previous studies have shown that *SUT1* is highly expressed in the developing seeds of different cereal crops including rice, barley and wheat. In rice, *SUT1* is expressed at similar levels from early to late stage of seed filling [20]. Consistently, antisense expression of *OsSUT1* resulted in impaired seed filling and retarded germination with no effect on photosynthesis in the flag leaf [21]. The *SUT1* of barley, however, exhibited its highest expression during the mid-stage of seed development. The transcripts of *OsSUT1* and *HvSUT1* are localized mainly in the maternal nucellar projections, aleurone tissues, and filial transfer cells that separate the endosperm cavity from the endosperm [6,20]. Furthermore, anti-OsSUT1 antibody was shown to bind to the plasma membranes of nucellar projections and aleurone tissues of developing wheat seeds [22]. As the maternal and filial tissues of cereal seeds lack symplastic connections; these results suggest the role of SUT1 in the post-phloem transport of sucrose from the maternal to the filial tissues of developing seeds.

Plant SUTs have been classified into five subfamilies, SUT1 to SUT5 [23]. Based on their significant similarity at the amino acid level, *OsSUT2* and *HvSUT2* are assigned to the SUT4 subfamily rather than to the monocot specific SUT3 or SUT5 subfamilies [9], and these genes are found to be expressed in both source and sink tissues [6,24]. The expression of *HvSUT2* in developing seeds was relatively predominant during the earlier and later stages [6], whereas that of *OsSUT2* was restricted only to the earlier stages [24]. In developing barley seeds, *HvSUT2* has been shown to have almost similar cellular localization as that of *HvSUT1*[6]. Recent proteomic and green fluorescent protein (GFP) fusion protein studies showed that some members of the SUT4 subfamily including HvSUT2 and OsSUT2, and those derived from other species such as Arabidopsis (AtSUT4), Lotus (LjSUT4) and poplar (PtaSUT4) are localized to the vacuolar tonoplast [25-27], suggesting that these proteins are involved in vacuolar storage of sucrose and its transport across the tonoplast to the cytosol. Consistently, a knockout mutation of *OsSUT2* and RNAi mediated suppression of *PtaSUT4* caused accumulation of sucrose in source leaves of rice and poplar, respectively, due to a decrease in sucrose transport from the vacuolar to the cytoplasmic compartment [27,28].

Plant SUTs play important roles in the allocation of photoassimilates in the form of sucrose from the source to sink tissues, and thereby determine seed yield. Identifying and characterizing new SUTs, thus, provide insights into the regulation of sucrose transport. Although wheat is one of the most economically important crops of the world, only one wheat *SUT* gene has been identified and characterized to date [8]. The present study identified a new *SUT*, designated as *TaSUT2*, and its homeologous from hexaploid wheat (*T. aestivum* cv. AC Andrew), and determined their subcellular localization and functionality as SUTs using a yeast heterologous system. Furthermore, the total expression of *TaSUT2* and the relative contribution of each of the three genomes were investigated in both source and sink tissues at different stages of development.

## Methods

### Plant growth conditions and tissue collection

Wheat plants (*T. aestivum* cv. AC Andrew) and the three diploid progenitors of hexaploid wheat, *T. urartu* (donor of A genome; accession # CN38564)*, Aegilops speltoides* (donor of B genome; accession # CN108020), and *Aegilops tauschii* (donor of D genome; accession # PI560538) were used for this study. Mature dry seeds were imbibed on a moist sterile Whatman #1 filter paper in a Petri plate (15 seeds per plate) in darkness for three days. Germinated seedlings were planted in 1-gallon pot (1 seedling per pot) containing Sunshine Mix #4 (LA4; Sungro Horticulture, Bellevue, WA, USA) and ~19 g mineral supplement (Cornell mixture; 100 g calcium carbonate, 150 g osmocote [18-6-12], 120 g superphosphate [0-45-0], 2 g fritted trace elements, 1.5 g chelated iron [13.2%], 0.7 g chelated zinc [14%]) at a depth of ~2 cm. The pots were then placed in a growth chamber at 18°C/14°C (day/night) under a 16/8 h photoperiod with cool white fluorescent (F96T12/CW/VHO; Sylvania, Danvers, MA, USA) light (175 μmol m^-2^ s^-1^) until harvest.

Young leaves harvested from 15- to 35-day-old plants of cv. AC Andrew and the three diploid progenitors were used for cloning the cDNAs of *TaSUT2*s. For all other studies, tissues were harvested at heading (eight days before anthesis) and at different days after anthesis (DAA). Vegetative tissues harvested at heading included flag leaf blade, flag leaf sheath and peduncle. To collect tissues at different DAAs, plants were tagged upon the first extrusion of the yellow anthers and this stage was considered as 0 DAA. The vegetative tissues described above along with developing spikes were harvested from individual primary or secondary tillers (one tiller per plant per replication; 3 replications) at 5, 10, 15, 20, 25 and 30 DAA. To minimize variations between samples, the seed and non-seed components including glumes, lemmas and paleas were harvested from the middle region of each spike (6–10 spikelets; 2–3 florets per spikelet). Tissues were frozen in liquid N_2_ immediately after harvesting and then stored at -80°C until further use.

### RNA extraction and cDNA synthesis

Total RNA was extracted from vegetative tissues (~100 mg fresh weight per sample) using RNeasy Plant Mini Kit (Qiagen, Hilden, Germany) as recommended by the manufacturer. Extraction of total RNA from developing seeds was performed as described before [29]. The RNA samples were then subjected to cDNA synthesis using the RevertAid™ H Minus First Strand cDNA Synthesis Kit (Fermentas, Glen Burnie, MD, USA) following the manufacturer’s protocol.

### Molecular cloning of *TaSUT2* cDNA

The cDNA samples derived from the young leaves of hexaploid wheat cv. AC Andrew were amplified using specific primers designed from the conserved coding regions of the previously identified *SUT2* genes of barley [GenBank:AJ272308] [6], maize [GenBank:AY639018] [30] and rice [GenBank:AY137242] [5]. The resulting PCR fragment was cloned into pGEM-T Easy vector (Promega, Madison, WI, USA) and sequenced. Gene specific primers were designed from the newly isolated partial fragment to identify the 5′ and 3′ end fragments with RACE-PCR using SMARTer RACE cDNA amplification kit (Clontech, Mountain View, CA, USA). Amplification products of the 5′ RACE and 3′ RACE were cloned into pGEM-T Easy vector (Promega) and then sequenced. Following end-to-end PCR the resulting DNA fragment was sequenced and then BLAST searched against the GenBank database.

### Identification of *TaSUT2* from the A, B and D genomes of hexaploid wheat

In order to identify the three homeologues of *TaSUT2* (*TaSUT2A*, *TaSUT2B* and *TaSUT2D*)*,* cDNA samples prepared from the young leaves of cv. AC Andrew and the three diploid progenitors of hexaploid wheat, *T. urartu* (A genome donor)*, Ae. speltoides* (B genome donor) and *Ae. tauschi* (D genome donor), were amplified using *TaSUT2* specific primers. The amplified fragments of *TaSUT2*, and *TuSUT2, AesSUT2* and *AetSUT2* were cloned into pGEM-T Easy vector (Promega) and then sequenced. The genomic origins of the resulting cDNAs of *TaSUT2* were determined by comparing their respective nucleotide sequences with those derived from the three diploid progenitors.

### Sequence and phylogeny analysis

Sequence homology of *TaSUT2A, TaSUT2B* and *TaSUT2D* with other cereal *SUT* genes was analyzed by using DNAMAN (http://www.lynnon.com/pc/alignm.html), and their respective coding sequences were identified by using Open Reading Frame (ORF) finder (http://www.ncbi.nlm.nih.gov/gorf/gorf.html). The ORFs of the three *TaSUT2* homeologues were translated into amino acid sequences by using the JustBio translator tool (http://www.justbio.com/index.php?page=hosted-tools) and then subjected to protein sequence homology analysis using DNAMAN. The molecular masses of TaSUT2A, TaSUT2B and TaSUT2D were determined by using protein molecular weight calculator software (http://www.sciencegateway.org/tools/proteinmw.htm). Membrane topology of the three TaSUT2s was predicted by using TMpred software (http://www.ch.embnet.org/software/TMPRED_form.html) [31]. In order to determine the phylogenetic relationship of the three TaSUT2s with other known plant SUTs, their deduced amino acid sequences were aligned with those corresponding to 40 SUTs derived from both monocot and dicot species using ClustalW program (http://www.ebi.ac.uk/Tools/msa/clustalw2) [32]. Unrooted neighbor-joining phylogenetic tree was generated using Molecular Evolutionary Genetic Analysis (MEGA, version 5) software (http://www.megasoftware.net) [33] with a Poisson correction model and a 500 replicate bootstrap method of phylogeny test. In order to predict the chromosomal location of the three homeologues of *TaSUT2*, the International Wheat Genome Sequencing Consortium (IWGSC) Survey Sequence Repository was searched for contiguous DNA sequences (contigs) with the cDNA sequences of *TaSUT2A*, *TaSUT2B* and *TaSUT2D* as queries.

### Heterologous expression of TaSUT2 in yeast

The functionality of the three *TaSUT2* homeologues in uptaking sucrose was examined by heterologous expression of their respective cDNAs in the cells of mutant SUSY7/*ura3* yeast (*Saccharomyces cerevisiae*) strain [34], which cannot utilize external sucrose as the sole carbon source since it lacks the extracellular invertase. To this end, full length coding sequences of *TaSUT2A*, *TaSUT2B* and *TaSUT2D* were cloned into the yeast expression vector pDR196 [35], producing pDR196-TaSUT2 constructs. Following verification of the sequence of each insert, the constructs were transformed into SUSY7/*ura3* cells, which were subsequently grown for 10 days at 30°C on synthesis complete (SC) media containing 2% sucrose at pH 5.2 as a sole source of carbon. All the transformants were also cultured on a medium containing 2% glucose at pH 5.8 as a sole source of carbon. The SC media were prepared as described before [36], and the SUSY7/ura3 cells transformed with pDR196 harboring the previously characterized high affinity potato SUT1 [37] and the vector control (empty pDR196 vector) were used as positive and negative controls, respectively.

### Transient expression of TaSUT2-YFP fusion protein

To determine the localization of TaSUT2 at subcellular level, its cDNA was first subcloned in frame with the N terminus of the yellow fluorescent protein (YFP) in the pEarleyGate 101 vector [38]. The TaSUT2-YFP fusion protein was then transiently expressed in onion (*Allium cepa*) epidermal cells via gold particles bombardment using a Helium Biolistic Particle Delivery system (PDS-1000, Bio-Rad, Hercules, CA, USA). Images of fluorescent cells were captured (1555-ms exposure time) with Axio Imager Z1 microscope (Carl Zeiss, Jena, Germany), and analyzed with AxioVision software (Carl Zeiss).

### Real time qPCR assay

For real time qPCR analysis, primers specific to TaSUT2, 5′-TACGGAGTCCTGCTCTGTCA-3′/5′-CTCGTCGCTTCCGAAAGTA-3′, and Taβ-actin (used as a reference gene), 5′-CCTTCCACATGCCATCCTTC-3′/5′-GCTTCTCCTTGATGTCCCTTAC-3′, were designed by using Primer3 software (http://frodo.wi.mit.edu/primer3/). To allow the detection of all transcripts derived from the three genomes, the TaSUT2 primers were designed from a region conserved across the three homeologues. Real time qPCR assays were performed using Maxima SYBR Green/ROX qPCR Master Mix (Fermentas). The reaction mixture contained 2 μL of cDNA (100 ng/μl), 10 μL of Maxima SYBR Green/ROX qPCR Master Mix, 0.6 μL of forward primer (10 μM; 300 nM final concentration), 0.6 μL of reverse primer (10 μM; 300 nM final concentration) and 6.8 μL of water, with a total reaction volume of 20 μL. Amplification and fluorescent signal detection was performed on a Mx3000P real time PCR System (Stratagene, La Jolla, CA, USA) using the following thermocycling conditions: initial denaturation and DNA polymerase activation at 95°C for 10 min, followed by 40 cycles of denaturation at 95°C for 15 s, annealing and extension at 60°C for 1 min in 96-well optical reaction plates covered with optical caps (Bio-Rad). The relative transcript level of *TaSUT2* was determined by 2^-ΔΔCt^[39].

### Genome specific semi quantitative PCR

**Figure 1:**
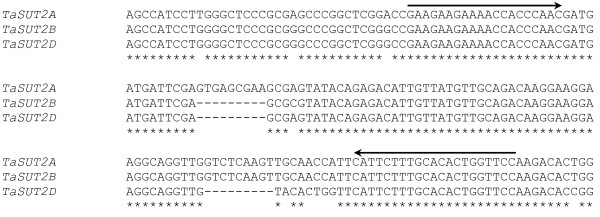
**Alignment of the partial 3’ UTR sequences of the three *TaSUT2 *homeologues.** Forward and reverse primers that span the polymorphic 3′ UTR regions and used to amplify three distinct DNA fragments corresponding to the amplicons of each homeologue are shown in arrows.

Forward and reverse primers that span polymorphic regions in the 3′ untranslated (UTR) of *TaSUT2A*, *TaSUT2B* and *TaSUT2D* (Figure 1) were used to amplify three distinct fragments corresponding to each homeologue from the cDNA samples. The PCR products were separated vertically on a 12% polyacrylamide gel (acrylamide:bisacrylamide ratio of 29:1). Following gel staining with ethidium bromide, the DNA fragments were visualized and the gel images captured using Gel Doc XR system (Bio-Rad). Band intensity of the PCR products corresponding to the amplicon of each homeologue was determined using Quantity One software (Bio-Rad) and then normalized to the background signal.

### In situ hybridization

Transverse sections of 4 DAA seeds and the youngest fully expanded leaf from 1-month-old plant were fixed in 4% paraformaldehyde (w/v) in 1X Potassium Buffer Saline (PBS) and then embedded in paraffin Paraplast. Digoxigenin (DIG)-labelled riboprobes were synthesized by *in vitro* transcription from a pGEM-T Easy vector (Promega) harboring a 605 bp cDNA fragment conserved across the three *TaSUT2*s using T7 and SP6 RNA polymerases with DIG-RNA labeling kit (Roche Diagnostics, Indianapolis, IN, USA). Both the sense and antisense probes were subjected to alkaline hydrolysis at 65°C to obtain approximately 150 bp fragments and then stored at −80°C until further use. Following pre-hybridization treatments, tissue sections (10 μm) were hybridized and then subjected to post-hybridization treatments, washes and antibody treatment as described previously [40]. Afterwards the sections were stained overnight in Western Blue (Promega) and then visualized under microscope (DC500; Leica, Wetzlar, Germany).

## Results

### Molecular cloning of *TaSUT2*

Amplification of the cDNA samples prepared from the young leaf tissues of *T. aestivum* cv. AC Andrew with forward and reverse primers designed from the conserved regions of *SUT2* homologs of barley (*HvSUT2*), rice (*OsSUT2*) and maize (*ZmSUT2*) produced a specific partial cDNA fragment of *TaSUT2*. RACE-PCR with gene specific primers derived from the partial cDNA fragment followed by end-to-end PCR produced a putative coding *TaSUT2* sequence of 1518 bp in length. Searching the GenBank database with the coding DNA sequence of *TaSUT2* revealed that *TaSUT2* has 93% identity with *HvSUT2*, and 80% identity with both *OsSUT2* and *ZmSUT2*.

### Identification of *TaSUT2* from the three diploid progenitors of hexaploid wheat

Amplification of cDNA samples derived from the leaf tissues of each diploid progenitor of hexaploid wheat, *T. urartu* (A genome donor)*, Ae. speltoides* (B genome donor) and *Ae. tauschi* (D genome donor) with forward and reverse primers specific to the *TaSUT2* generated by RACE-PCR produced DNA fragments corresponding to the putative *SUT2* of each progenitor. Nucleotide sequencing followed by analysis with ORF finder indicated that *TuSUT2A*, *AesSUT2B* and *AetSUT2D* genes have coding DNA sequences of 1518, 1518 and 1524 bp, respectively, encoding 506, 506 and 508 amino acids that exhibited over 99% similarity to one another.

### Identification of *TaSUT2s* from the A, B and D genomes of hexaploid wheat

Sequencing of multiple colonies generated by transformation of *E.coli* (DH5α) cells with the putative *TaSUT2* generated by RACE-PCR revealed the presence of three homeologues of *TaSUT2* in hexaploid wheat. Comparison of the sequences of their coding regions and the respective untranslated fragments with those obtained from each of the three diploid progenitors enabled us to identify their genomic origin. Nucleotide sequences of the coding region and untranslated fragments of *TaSUT2A* showed 100% identity with that of *TuSUT2*. Similarly, the DNA sequences of the coding and untranslated fragments of *TaSUT2D* showed 100% identity with that of *AetSUT2*. Whereas, the *TaSUT2B* exhibited less than 100% (99.5%) identity with the coding DNA sequence of *AesSUT2* due to few base substitutions.

### Chromosomal location of the three *TaSUT2* homeologues of hexaploid wheat

BLAST searching the IWGSC Survey Sequence Repository with DNA sequences of *TaSUT2A*, *TaSUT2B* and *TaSUT2D* produced seven contigs from A, B and D genomes of chromosome 5 as having the best hits with E-values less than 2e^-42^ and showing over 81% identity with the respective *TaSUT2* cDNAs for matching sequences ranging from 168 to 741 bp (Additional file 1: Table S1). These results allowed us to predict chromosomes 5A, 5B and 5D as the locations where the three homeologues of *TaSUT2* reside in the wheat genome.

### Sequence and phylogeny analysis of the three TaSUT2 genes of hexaploid wheat

**Figure 2:**
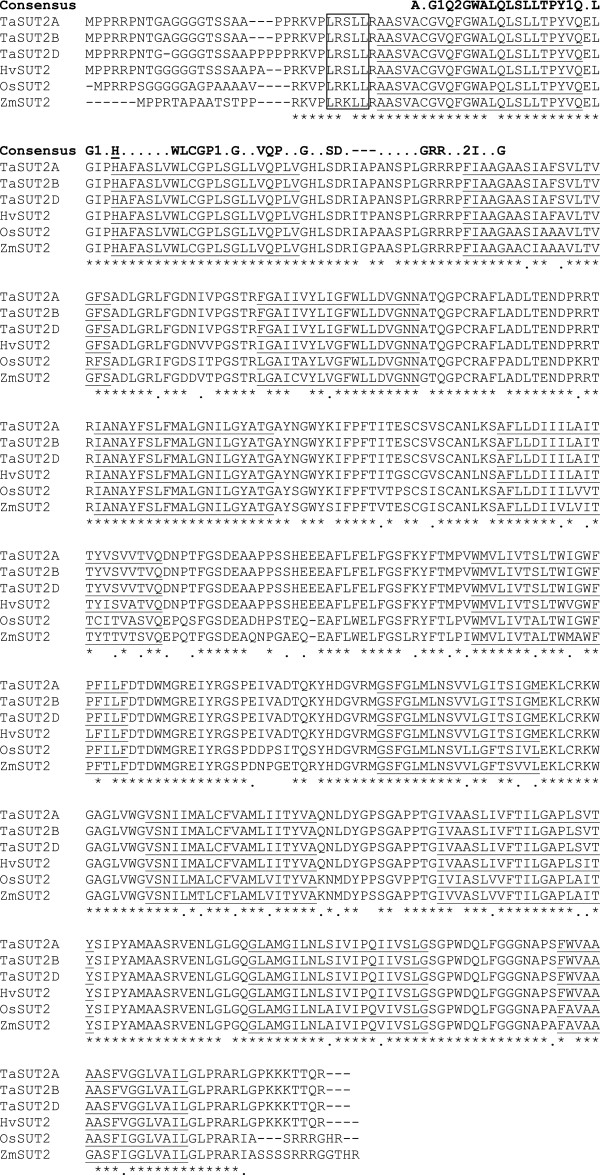
**Alignment of the amino acid sequences of TaSUT2s with other cereal SUT2s.** Amino acid sequences of TaSUT2A, TaSUT2B and TaSUT2D were aligned using the DNAMAN program with SUT2s of barley (*Hordeum vulgare*; HvSUT2), rice (*Oryza sativa*; OsSUT2) and maize (*Zea mays*; ZmSUT2). Similarity in amino acids across all the sequences (92.6%) is indicated by stars. The amino acid sequences were also compared with the CONSENSUS sequence derived from the highly conserved region of functional plant SUTs, where 1 = I, L or V; 2 = F, W or Y. The putative vacuolar targeting dileucine motif is shown in a box, and the histidine residue that appears to be located at or conformationally linked to sucrose binding site of SUTs is shown in bold and underlined. The 12 transmembrane helices, coined as distinct characteristic features of all members of the GPH cation symporter family to which all the known plant SUTs belong, are underlined.

**Figure 3:**
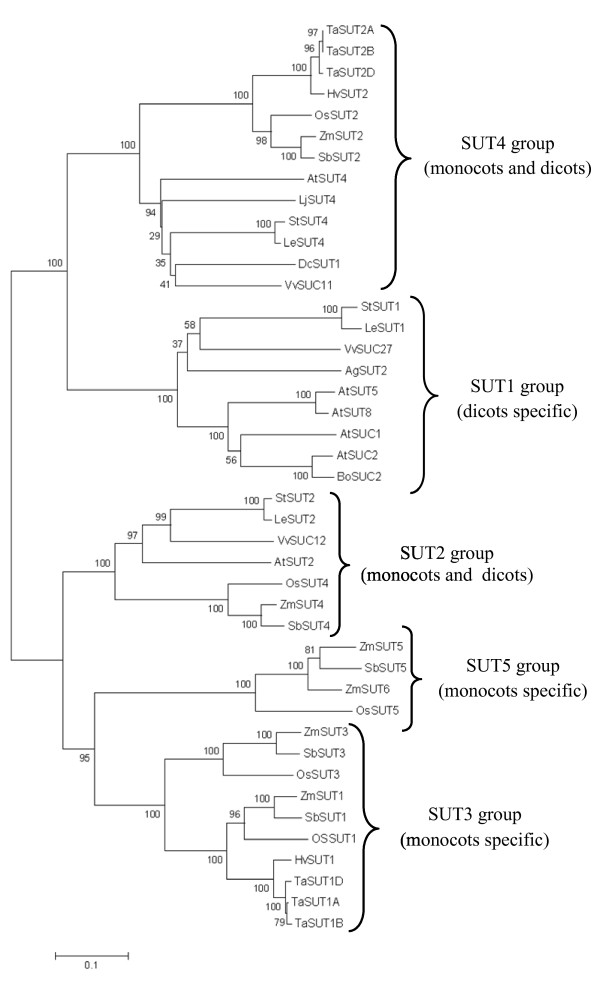
**Phylogenetic relationships of TaSUT2 with other plant SUTs.** Phylogenetic tree was generated based on amino acid sequence similarity of *TaSUT2A*, *TaSUT2B* and *TaSUT2D* with that of SUTs identified from representative monocotyledonous and dicotyledonous species using MEGA program [33]. The evolutionary history was inferred using the Neighbor-Joining method. The percentage of replicate trees in which the associated taxa clustered together in the bootstrap test (500 replicates) is shown next to the branches. The evolutionary distances were computed using the Poisson correction method and are in the units of the number of amino acid substitutions per site. The analysis involved amino acid sequences of 40 SUTs: *Apium graveolens,* AgSUT2, AF167415; *Triticum aestivum*, TaSUT1A, AAM13408; TaSUT1B, AAM13409.1; TaSUT1D, AAM13410.1; *Hordeum vulgare*, HvSUT1, CAJ20123.1; HvSUT2, CAB75881.1; *Oryza sativa*, OsSUT1, BAI83443.1; OsSUT2, BAC67163.1; OsSUT3, BAB68368.1; OsSUT4, BAC67164.1; OsSUT5, BAC67165.1; *Zea mays,* ZmSUT1, NP_001104840; ZmSUT2, AAT51689; ZmSUT3, ACF86653.1; ZmSUT4, AATS91375.1; ZmSUT5, ACF85284.1; ZmSUT6, ACF86653.1; *Solanum tuberosum*, StSUT1, CAA48915.1; StSUT2, AAP43631.1; StSUT4, AAG25923.2; *Arabidopsis thaliana*, AtSUC1, CAA53147.1; AtSUC2, CAA53150.1; AtSUT2, AAC32907.1; AtSUT4, AAG09191.1; AtSUT5, BAB11624.1; AtSUT8, AAC69375.1; *Lycopersicon esculentum*, LeSUT1, CAA57726.1; LeSUT2, AAG12987.1; LeSUT4, AAG09270.1; *Vitis vinifera,* VvSUC11, AAF08329.1; VvSUC12, AAF08330.1; VvSUC27, AAF08331.1; *Daucus carota*, DcSUT1A, CAA76367.1; *Sorghum bicolor*, SbSUT1, ACY69230.1; SbSUT2, XX_00243677.1; SbSUT3, XP_002467275.1; SbSUT4, EES06059.1; SbSUT5, XP_002454058.1; *Brassica oleracea*, BoSUC2, AAL58072.1; *Lotus japonica*, LjSUT4, AJ538041.

Comparative homology analysis of the coding DNA sequences of *TaSUT2A*, *TaSUT2B* and *TaSUT2D* genes by using DNAMAN showed that they share 99.1% identity one another, and 90.1% identity with *SUT2*s of rice, barley and maize. Translation of their predicted ORFs using the JustBio translation tool showed that *TaSUT2A*, *TaSUT2B* and *TaSUT2D* cDNAs encode 506, 506 and 508 amino acids with estimated molecular masses of 53.87, 53.87 and 53.92 kDa, respectively. The predicted amino acid sequences of TaSUT2A, TaSUT2B and TaSUT2D exhibited over 99.3% identity one another and 92.6% identity with those of HvSUT2, OsSUT2 and ZmSUT2 proteins (Figure 2). BLAST search analysis against the GenBank database showed that the three homeologues of *TaSUT2* are new members of the GPH cation symporter family to which all other plant SUTs belong. Protein topology prediction using the TMpred program indicated that all the three proteins contain 12 transmembrane helices (Figure 2). Furthermore, the three SUT2s of wheat contain the consensus sequence derived from the highly conserved region of functional plant SUTs [5] and the histidine residue that appears to be localized at or associated conformationally with sucrose binding sites of SUTs (Figure 2). Generation of a phylogenetic tree using the MEGA software based on the amino acid sequences of TaSUT2A, TaSUT2B and TaSUT2D, and other representative SUTs from both monocot and dicot species showed that the three TaSUT2s are members of the SUT4 subfamily (Figure 3), which mainly contains tonoplast localized SUTs. Consistently, the TaSUT2s contain the putative vacuolar targeting dileucine motif (LXXLL) found in the cytoplasmic N-terminus of all members of the SUT4 subfamily (Figure 2).

### Functionality of the three TaSUT2s proteins of hexaploid wheat

**Figure 4:**
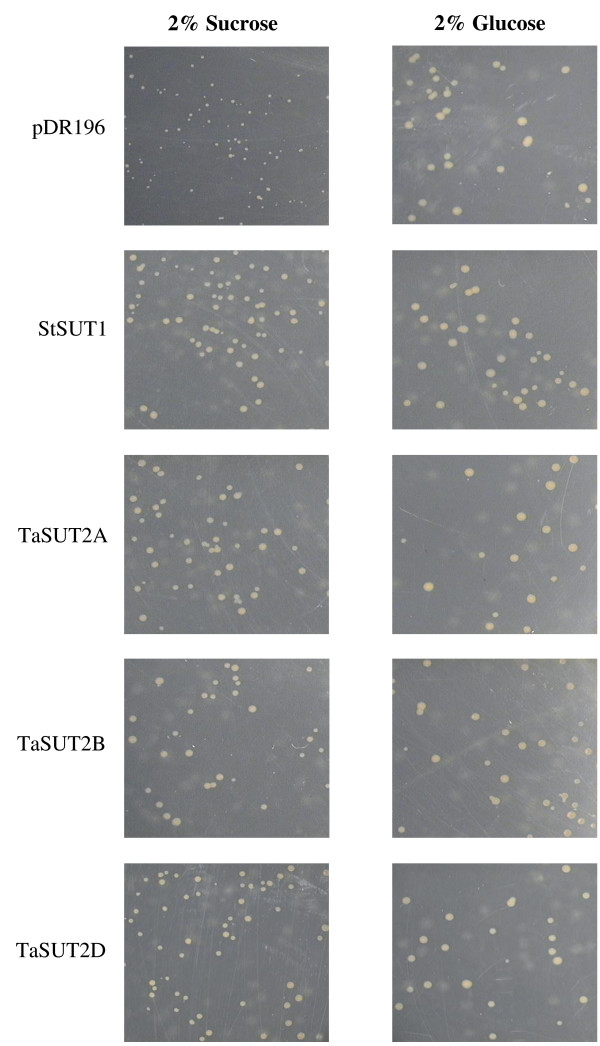
**Growth of SUSY7/ura3 yeast cells transformed with pDR196-TaSUT2s on media containing 2% (w/v) sucrose and glucose.** SUSY7/ura3 cells transformed with the empty pDR196 vector (negative control), pDR196-StSUT1 (positive control), pDR196-TaSUT2A, pDR196- TaSUT2B and pDR196-TaSUT2D. Slower growth of SUSY7/ura3 cells transformed with the empty pDR196 vector was observed on sucrose containing medium.

The functionalities of the three TaSUT2s were determined *in vitro* by complementation analysis of the mutant SUSY7/*ura3* yeast strain, which normally cannot grow on sucrose containing medium. The SUSY7/*ura3* cells transformed with pDR196 containing each of the three *TaSUT2* homeologues (*TaSUT2A, TaSUT2B* and *TaSUT2D*) and the *StSUT1* (used as a positive control) exhibited faster growth on media containing sucrose as a sole source of carbon when compared to the corresponding cells transformed with the vector control (Figure 4). Further analysis with glucose containing media revealed that SUSY7/*ura3* cells transformed with the *TaSUT2* and *StSUT1*, and the vector control exhibited comparable growth rates on glucose as a sole source of carbon (Figure 4).

### Subcellular localization of TaSUT2

**Figure 5:**
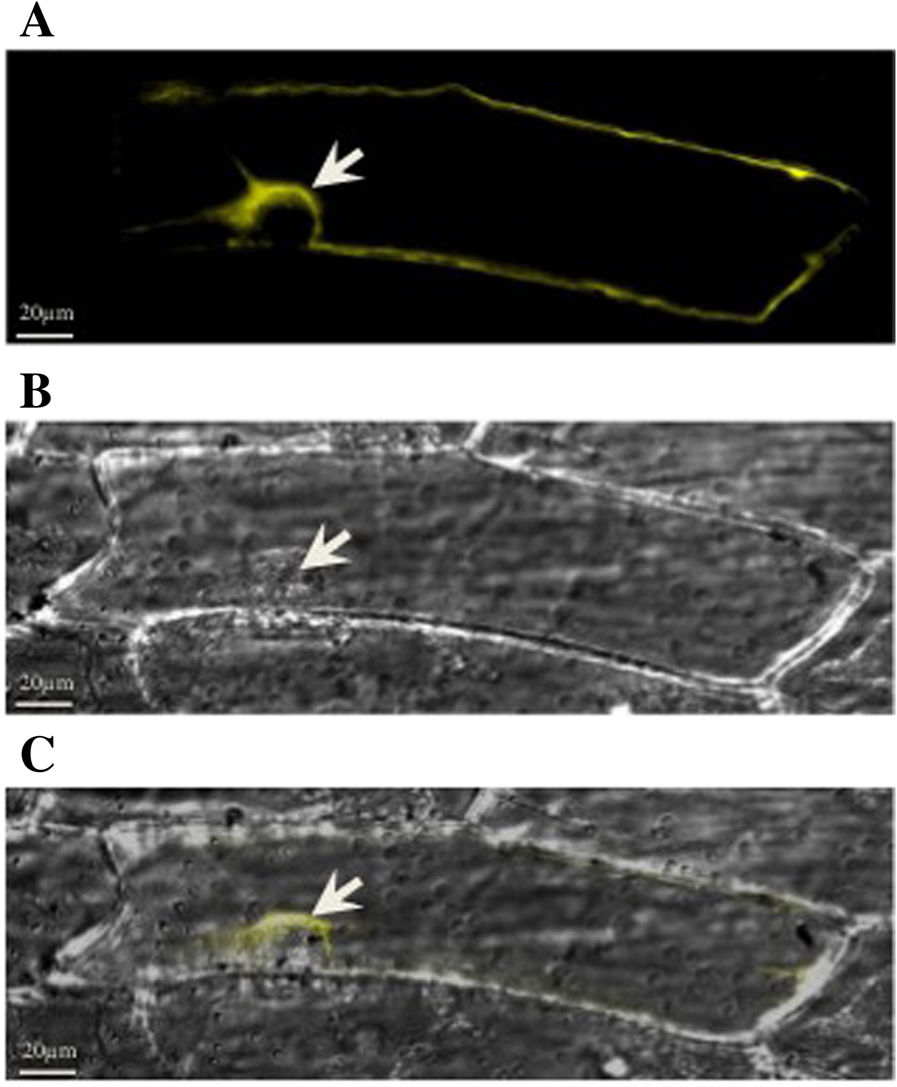
**Subcellular localization of TaSUT2 transiently expressed in onion epidermal cells.** Localization of the TaSUT2-YFP fusion protein to the tonoplast is shown by the white arrow **(A)**. Differential interference contrast (DIC) image of the same onion epidermal cell **(B)**; the white arrow indicates the nucleus of the cell. Merged images of A and B to co-localize the TaSUT2-YFP to the tonoplast **(C)**. No YFP-fluorescing signal was detected in the negative control (data not shown).

Analysis of the localization of TaSUT2 at the subcellular level through transient expression of a TaSUT2-YFP fusion protein in onion epidermal cells revealed strong YFP-fluorescing signal on the inside of the nucleus around a structure that appears to be the vacuole (Figure 5), indicating that TaSUT2 is localized to the tonoplast rather than to the plasma membrane.

### Expression of *TaSUT2* in developing seeds

**Figure 6:**
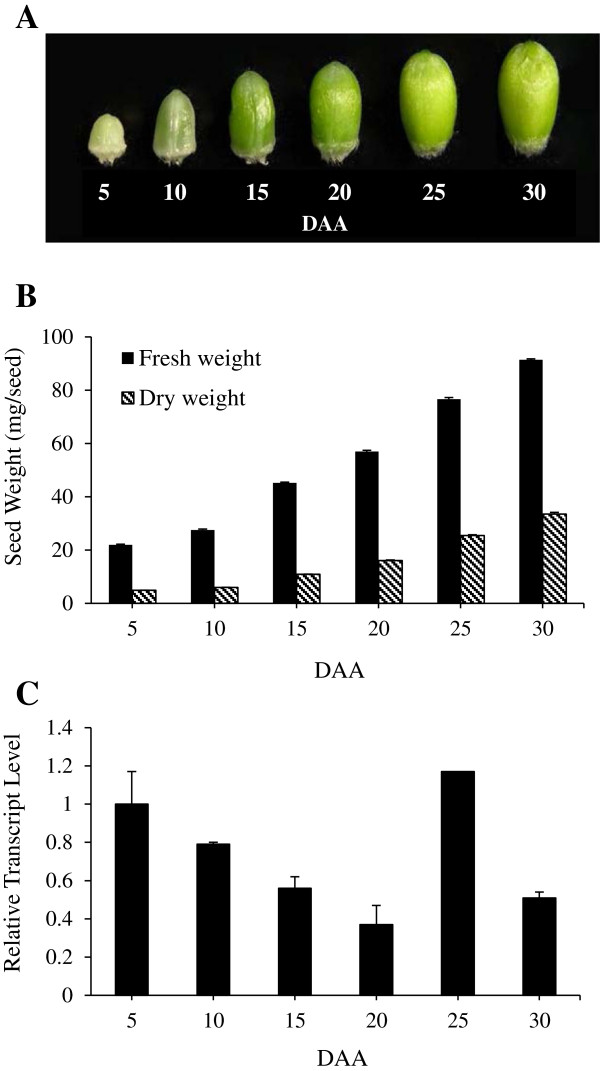
**Developing wheat seeds and the total expression of *TaSUT2.*** Seeds at 5, 10, 15, 20, 25 and 30 DAA **(A)** and their fresh and dry weights **(B)**. Data are means ± SE, n = 20. Relative abundance of *TaSUT2* transcript in the same wheat seed samples **(C)**. Transcript levels were determined after normalization with actin as the reference gene, and then expressed relative to that in 5 DAA seed samples, which was arbitrarily set to a value of 1. Data are means ± SE, n = 2 to 3.

The total expression of *TaSUT2* was investigated in developing seeds at 5, 10, 15, 20, 25 and 30 DAA using real time qPCR. Initiation of fresh and dry matter accumulation in the seeds by 5 DAA was associated with higher transcript abundance of *TaSUT2* (Figure 6). Further increase in fresh and dry weights as the seed develops through 20 DAA was, however, accompanied by a gradual decline in the abundance of *TaSUT2* transcripts. As seed growth continued to increase from 20 to 25 DAA, the level of *TaSUT2* transcripts increased 3-fold, attaining a level similar to that observed in 5 DAA seeds. Seed growth from 25 to 30 DAA was characterized by a decline in the abundance of *TaSUT2* transcripts.

### Expression of *TaSUT2* in source tissues

**Figure 7:**
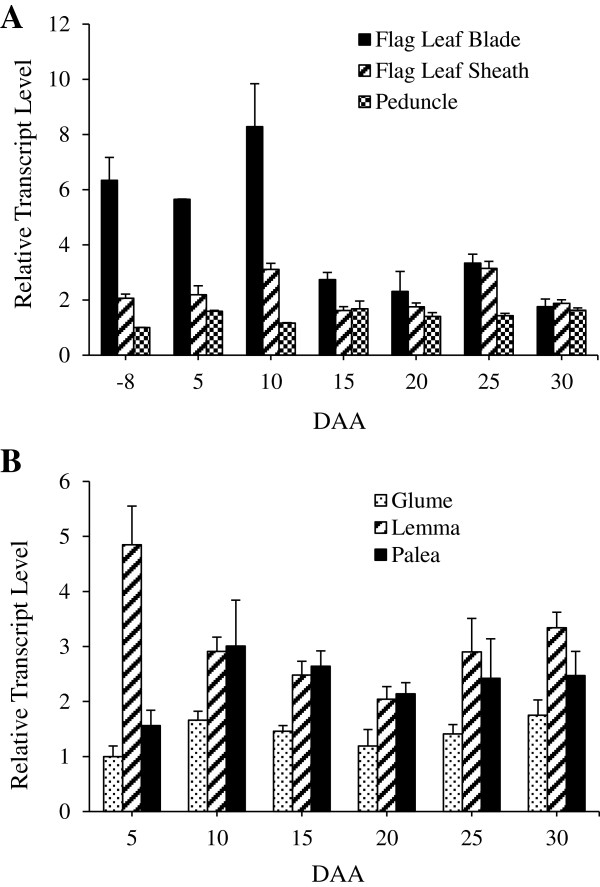
**Total expression of *TaSUT2 *in the source tissues during seed development.** Relative transcript abundance of *TaSUT2* in flag leaf blade, flag leaf sheath and peduncle **(A)** and glume, lemma and palea **(B)** tissues during wheat seed development (−8, 5, 10, 15, 20, 25 and 30 DAA). Transcript levels were determined after normalization with actin as the reference gene, and then expressed relative to that in −8 DAA peduncle **(A)** and 5 DAA glume **(B)** samples, which were arbitrarily set to a value of 1. Data are means ± SE, n = 2 to 3.

The total expression of *TaSUT2* was also investigated in the source tissues including flag leaf blade, flag leaf sheath and peduncle (at heading and at 5, 10, 15, 20, 25 and 30 DAA), and non-foliar tissues of florets including glume, lemma and palea (at 5, 10, 15, 20, 25 and 30 DAA). The transcripts of *TaSUT2* were detected in all tissues examined (Figure 7A, B). The level of *TaSUT2* transcripts in the flag leaf blade was relatively higher at heading and during the early periods of seed development (5 to 10 DAA), after which it showed a 5-fold decrease and remained at a similar level through 30 DAA (Figure 7A). Expression of *TaSUT2* in the flag leaf sheath and peduncle tissues was almost similar across all the stages of seed development examined in this study, except that its level in the peduncle was slightly lower at the time of heading. The non-foliar lemma and palea tissues of developing florets exhibited similar abundance of *TaSUT2* transcripts, although substantially higher abundance was evident in the lemma at the early stage (5 DAA; Figure 7B). The pattern of glume-derived *TaSUT2* transcript abundance was almost similar to that observed in the lemma and palea tissues, but at a relatively lower level.

### Amplification of fragments unique to each homeologue of *TaSUT2*

The high percentage of nucleotide identity (~99%) among the three homeologues of *TaSUT2* could not allow us to design coding region-derived primers that are able to separately amplify amplicons of each homeologue. BLAST searching the coding DNA sequences of *TaSUT2A*, *TaSUT2B* and *TaSUT2D* against the IWGSC Survey Sequence Repository revealed contigs derived from the A, B and D genomes of wheat that contain a portion of the coding sequence (with over 99% sequence identity) and the respective 3′ UTR region of each homeologue. Alignment of the 3′ UTR sequences derived from the three genomes revealed a polymorphic region that can produce a unique amplicon for each homeologue (134 bp for *TaSUT2A*; 125 bp for *TaSUT2B* and 116 bp for *TaSUT2D*; Figure 1). Separation with polyacrylamide gel of the PCR products amplified from the cDNA samples of cv. AC Andrew tissues with a primer set designed to span the polymorphic 3′ UTR regions (Figure 1) produced three distinct DNA fragments corresponding to the amplicons derived from each genome (Additional file 2: Figure S1).

### Genomic contribution to the total expression of *TaSUT2*

**Figure 8:**
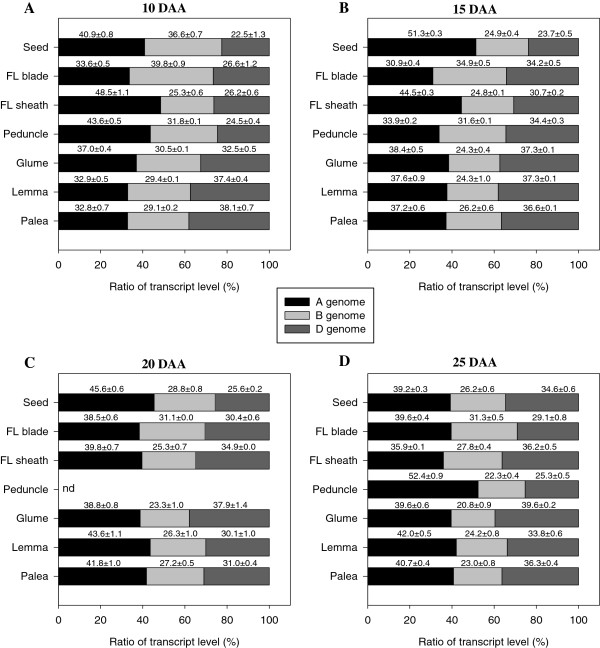
**Genomic contribution to the total expression of *TaSUT2*.** Percent of transcript contribution from each genome to the total expression of *TaSUT2* in the seed and source tissues during the rapid seed filling phase, 10 DAA **(A)**, 15 DAA **(B)**, 20 DAA **(C)** and 25 DAA **(D)**. FL, flag leaf; nd, not detected.

Transcript contribution of each genome to the total expression of *TaSUT2* was examined in both sink and source tissues during the rapid seed filling period, from 10 DAA to 25 DAA, using semi-quantitative RT-PCR. Our results showed that genomic contribution varies with tissues and stages (Figure 8). In the seed, genome A contributes the most; however, its contribution varies with stage (39% to 51%). With respect to the vegetative source tissues, genome B is the major contributor (40%) in the flag leaf blade at 10 DAA followed by genome A (34%) and then by D (27%). As the flag leaf blade develops from 10 to 15 DAA, both B and D genomes contribute equally at a level slightly higher than that of genome A, which appears to be a major contributor during the later stages, 20 to 25 DAA (39% to 40%). Although genome A contributes the most in the flag leaf sheath from 10 to 20 DAA (40% to 49%), both A and D genomes contribute equally at a level higher than that of genome B at 25 DAA. In the peduncle, genome A contributes the most at 10 DAA and 25 DAA (44% to 52%), however, the three genomes appear to contribute almost equally at 15 DAA. With respect to the non-seed source tissues of the spike, the contribution of genome A is predominant in the glume at 10 DAA, but both A and D genomes contributes almost equally, each at a higher level than that of B, in the subsequent stages. In the lemma and palea tissues, genome D has the most contribution at 10 DAA (38%), while both A and D genomes contribute equally (at higher level than that of B) at 15 DAA. From 20 to 25 DAA, genome A is the major contributor in these tissues followed by D and then by B. When all tissues and developmental stages are taken into account, the average contribution of genome A (40%) is higher than that of genome D (32%) and genome B (28%).

### Cellular localization of *TaSUT2* transcripts in developing seeds and source leaf blade

**Figure 9:**
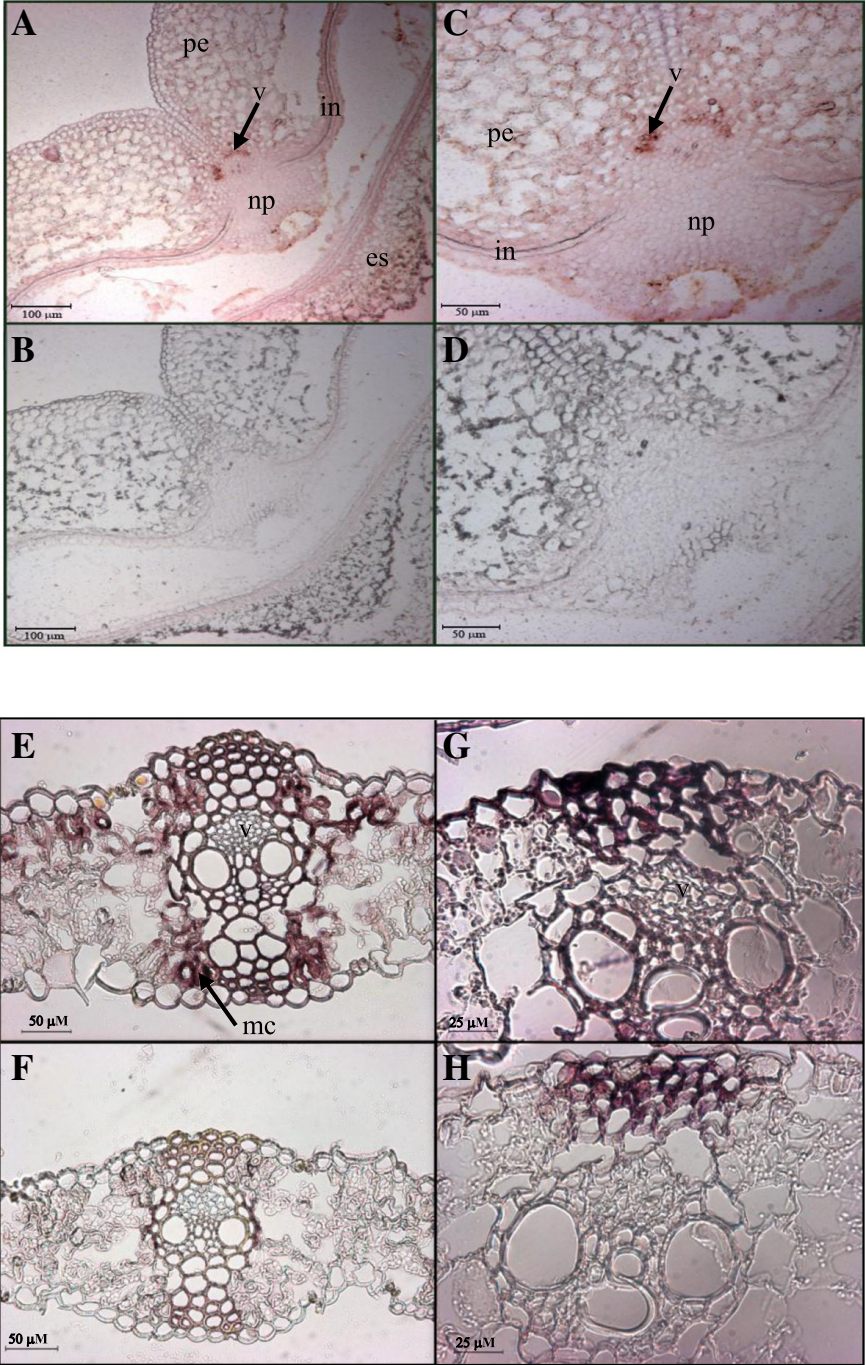
**Cellular localization of *TaSUT2 *transcripts in wheat seeds and leaves.** Transverse sections of the middle portion of 4 DAA wheat seeds probed with digoxygenin-labeled antisense **(A, C)** and sense **(B, D)***TaSUT2* riboprobes. The *TaSUT2* transcripts are mainly localized to the vein (see the red-brown staining indicated by the arrow in **A, C**). Weak signal (light pink staining) was also detected in the tip of the nucellar projection and in the integument **(A**, **C)**. **A** and **B**, and **C** and **D** are at 5X and 10X magnifications, respectively. Transverse sections of the youngest fully expanded leaf of 1-month-old wheat plant probed with digoxygenin-labeled antisense **(E, G)** and sense **(F, H)***TaSUT2* riboprobes. The *TaSUT2* transcripts are mainly localized to the subepidermal mesophyll cells (see the red-brown staining indicated by the arrow in **E)**. Weak signal (light pink staining) was also detected in the vein **(E**, **G)**. **E** and **F**, and **G** and **H** are at 10X and 20X magnifications, respectively. Transverse sections hybridized with the control sense probe **(B, D** and **F, H)** produced no signal. v, vein; np, nucellar projection; in, integument; es, endosperm; pe, pericarp; mc, mesophyll cells.

Localization of *TaSUT2* transcripts at the cellular level was examined in developing seeds and source leaf blade by *in situ* hybridization using a probe synthesized from the 605 bp coding DNA fragment conserved across the three *TaSUT2* homeologues so as to allow the detection of all transcripts from the three genomes. A strong signal of *TaSUT2* transcripts was mainly localized in the vascular vein of developing seeds (Figure 9A, C) and subepidermal mesophyll cells of the leaf blade (Figure 9E, G).

## Discussion

Starch deposition in the seeds of cereal crops is determined at least partly by the translocation of sucrose from the source tissues. Sucrose transport across the cellular membrane barriers is mediated by SUTs, and genes encoding these proteins have been identified and functionally characterized from a number of cereal crops including rice, maize and barley [23]. To date, however, only one *SUT* (*TaSUT1*) has been identified and characterized in hexaploid wheat [8,15,41]. To gain better insights into the regulation of the partitioning of assimilates from source tissues to developing seeds in wheat, it is important to isolate and characterize more SUTs. To this end, in the present study, we identified the three homeologues of a new *SUT* gene, designated as *TaSUT2*, and investigated their functionality, subcellular localization, and spatiotemporal and genome specific expression patterns. The *TaSUT2A*, *TaSUT2B* and *TaSUT2D* genes have respective ORFs of 1518 bp, 1518 bp and 1524 bp encoding proteins with 506, 506 and 508 amino acids, respectively (Figure 2), with estimated molecular masses of ~54 kDa. Similar to that observed in the three cDNAs of *TaSUT1*[8], the *TaSUT2A, TaSUT2B* and *TaSUT2D* genes differ mainly in the 3’ UTR region (Figure 1).

Several lines of evidence from sequence and phylogeny analyses suggest that the newly identified homeologues of *TaSUT2* encode putative SUTs. Firstly, their cDNA and deduced amino acid sequences show very high sequence homology with *SUT2*s (90%) and the corresponding proteins (93%), respectively, of rice, barley and maize (Figure 2). Secondly, their proteins contain the 12 transmembrane helices (Figure 2), coined as distinct characteristic features of all members of the GPH cation symporter family to which all the known plant SUTs belong [10,11], and the consensus sequence derived from the highly conserved region of functional SUTs (Figure 2) [5]. Thirdly, the histidine residue that is conserved across all known plant SUTs and appears to be localized at or associated conformationally with sucrose binding site of SUTs [12] is present in the first extracellular loop of TaSUT2s (His-61 in TaSUT2A and TaSUT2B, and His-63 in TaSUT2D; Figure 2). The significant similarity exhibited by partial cDNA sequences of *TaSUT2A*, *TaSUT2B* and *TaSUT2D* with contigs derived from chromosomes 5A, 5B and 5D (Additional file 1: Table S1) suggests that these genes are located on chromosome 5; establishing a differential chromosomal location between *TaSUT1*, which resides on chromosome 4 [8], and *TaSUT2*.

Phylogenetically, the TaSUT2A, TaSUT2B and TaSUT2D proteins are grouped into the SUT4 subfamily that includes low affinity and tonoplast localized dicot SUT4s and monocot SUT2s rather than into the monocot specific SUT3, which includes the TaSUT1, and SUT5 subfamilies (Figure 3) [42]. Furthermore, TaSUT2s contain the putative vacuolar targeting dileucine motif (LXXLL) found in the cytoplasmic N-terminus of all members of the SUT4 subfamily, except for AtSUT4 which instead has KRVLL [43]. Consistently, our subcellular localization analysis of TaSUT2 using YFP fusion protein showed that it is a tonoplast localized SUT (Figure 5). The low (< 45%) similarity of TaSUT2A, TaSUT2B and TaSUT2D with the corresponding homeologues of TaSUT1 explains the phylogenetic distant relationship between TaSUT1 and TaSUT2 proteins. Likewise, HvSUT1 and HVSUT2 that share only 42% similarity between one another [6] are grouped into two different SUT subfamilies, SUT3 and SUT4, respectively (Figure 3) [9]. To date, the SUSY7/*ura3* mutant strain of yeast has been used as a tool of choice to study the biological functionalities of plant SUTs. This mutant strain of yeast cannot utilize external sucrose as its cytosolic and extracellular invertases are knocked out [44]. However, the expression of a plant sucrose synthase in its cytosol enables the mutant strain to grow on sucrose provided that SUT is expressed in its plasma membrane. Complementation of the SUSY7/*ura3* yeast cells with TaSUT2A, TaSUT2B and TaSUT2D enabled the yeast cells to uptake sucrose and grow on media containing sucrose as a sole carbon source (Figure 4). This result is inconsistent with TaSUT2’s localization to the tonoplast, as a plasma membrane-localized SUT is required to uptake sucrose from the medium. It is therefore likely that TaSUT2 is mislocalized to the plasma membrane in the heterologous system, and this mislocalization artifact led to complementation of the yeast SUSY7/*ura3* mutant. Similar results of complementation of the SUSY7/*ura3* yeast cells with tonoplast-localized HvSUT2 of barley [6], PtaSUT4 of populus [27] and expression-optimized OsSUT2 of rice [28] have been reported. However, heterologous expression of a plant SUT in yeast does not necessarily reveal their subcellular localization [25].

In order to gain insights into the physiological roles of *TaSUT2*, we analyzed its total expression in the source (flag leaf blade and sheath, peduncle, glume, lemma and plaea) and sink (developing seeds) tissues before and after seed formation (Figures 6 and 7). The expression of *TaSUT2* in seeds was relatively higher during the early (5–10 DAA) and late (25 DAA) stages of their development (Figure 6), whereas *TaSUT1* was shown to be highly expressed during the mid/rapid phase of seed filling (16–20 DAH) [8]. It is therefore likely that TaSUT1 and TaSUT2 play functionally and temporally distinct roles during seed development. Consistent with these results, the expression of *HvSUT2* was relatively higher during the earlier and later stages of seed development, where as that of *HvSUT1* was predominant by the mid-phase [6]. Localization of *TaSUT2* transcripts in the vein of developing seeds (Figure 9A, C) suggests that TaSUT2s are involved in the partitioning of sucrose between the vacuolar and cytoplasmic cell compartments in the vein.

Seed filling in wheat is supported mainly by photoassimilates derived from the flag leaf [45]. Thus, the relatively higher abundance of *TaSUT2* transcripts in the source flag leaf blade before anthesis and during the early stages (5–10 DAA) of seed development (Figure 7A) may suggest the role for TaSUT2 in intracellular partitioning of sucrose in the leaves, and thereby regulating the allocation of assimilates from leaf to seed tissues. The other known wheat *SUT* gene, *TaSUT1*, which is localized to the plasma membrane and involved in phloem loading, also exhibit expression in the flag leaf blade before and after heading, although its level after heading was lower than before heading [8]. This implies the complementarity of these two genes in regulating sucrose transport from source to sink tissues; the *TaSUT2* controlling the cytosolic sucrose homeostasis while the *TaSUT1* regulating sucrose loading to the phloem. Consistently, the tonoplast localized *SUT2*s of barley [6] and rice [24] are found to be expressed in the source leaf tissues. Furthermore, *OsSUT2* mutants of rice and poplar plants expressing RNAi-suppressed *PtaSUT4* showed increased accumulation of sucrose in their leaves, which in turn resulted in reduced plant height, tiller number, and seed weight in rice, and increased leaf to stem biomass ratio in popular [27,28]. The decline in the transcript abundance of *TaSUT2* in the flag leaf blade during the later stages of seed filling can be associated with the senescence of leaves and decreased accumulation of storage sucrose in the vacuole [19,46]. The localization of *TaSUT2* transcripts in the mesophyll cells of leaf blade (Figure 9E, G) might imply their significance in regulating intracellular sucrose partitioning within these cells of a leaf tissue.

Parts of the peduncle and leaf sheath tissues of wheat are exposed to incoming radiation, and thus can photosynthesize and produce photoassimilates that contribute approximately 9% to 12% of wheat seed dry matter [18,46]. It has also been shown that photosynthesis in the glume, lemma and palea tissues of a wheat floret contributes 10% to 44% of the photoassimilates destined to wheat seed [17,18]. Thus, the expression of *TaSUT2* in the peduncle and leaf sheath (Figure 7A), and the glume, lemma and palea of the spike (Figure 7B) likely suggests its involvement in the intracellular transport of sucrose from the vacuole to the cytoplasm in these tissues. In agreement with this, source tissues of cereals primarily store sucrose rather than transitory starch temporarily in their vacuoles [47-49]. It appeared from our genome specific semi-quantitative RT-PCR analysis that the contribution of the three wheat genomes to the total expression of *TaSUT2* varies with tissues and developmental stages (Figure 8), suggesting a specific spatio-temporal role for each *TaSUT2* homeologue in determining the activity of TaSUT2.

## Conclusions

This study demonstrated that *TaSUT2* encodes a new wheat SUT localized to the tonoplast, and phylogentically it belongs to the SUT4 subfamily that mainly contains vacuolar membrane SUTs. These results along with its spatiotemporal expression patterns at the gene level suggest that TaSUT2 is involved in the exchange of sucrose between the vacuolar and cytoplasmic cell compartments in both sink and source tissues. However, elucidating the definitive physiological role of *TaSUT2* requires mutational analysis for changes in sucrose transport activity, levels of metabolites in the sucrose-starch pathway, and overall seed yield and other yield parameters.

## Abbreviations

CC: Companion complex; DAA: Days after anthesis; DIG: Digoxigenin; GPH: Glycoside-pentoside-hexuronide; PBS: Potassium buffer saline; SE: Sieve elements; SUT: Sucrose transporter.

## Competing interests

The authors declare that they have no competing interests.

## Authors’ contributions

BA conceived and designed the experiments. BA and KD analyzed the data and wrote the manuscript. KD, SM and FG performed the experiments. ABB and CS contributed materials and provided input into the projects direction. All authors read and approved the final manuscript.

## Supplementary Material

Additional file 1: Table S1 Best hit contigs that exhibit significant identity with *TaSUT2* cDNAs. The International Wheat Genome Sequencing Consortium survey sequence repository was searched for contiguous DNA sequences with the cDNA sequences of *TaSUT2A*, *TaSUT2B* and *TaSUT2D* as queries. The significance of the alignment score was determined by E value. Click here for file

Additional file 2: Figure S1 Amplification of DNA fragments unique to each homeologue of TaSUT2. Polyacrylamide gel separation of the three distinct DNA fragments produced from tissues of cv. AC Andrew by a primer set designed to span polymorphic 3′ UTR regions of the three homeologues. Lanes 1, 2 and 3 shows PCR products corresponding to the amplicons of each homeologue, and lane 4 represents the DNA ladder. Click here for file
