# Supplementary material for: Identification and characterization of the three homeologues of a new sucrose transporter in hexaploid wheat (Triticum aestivum L.)
Source: BMC Plant Biol. 2013 Nov 16;13:181. doi: 10.1186/1471-2229-13-181 (PMC4225610; doi:10.1186/1471-2229-13-181)
Supplement: Additional file 1: Table S1 — Best hit contigs that exhibit significant identity with TaSUT2 cDNAs. The International Wheat Genome Sequencing Consortium survey sequence repository was searched for contiguous DNA sequences with the cDNA sequences of TaSUT2A, TaSUT2B and TaSUT2D as queries. The significance of the alignment score was determined by E value. [file 1471-2229-13-181-S1.pdf]

Table S1. Best hit contigs that exhibit significant identity with the coding DNA sequences of *TaSUT2*s.

Query sequence[a]										
TaSUT2A			*TaSUT2B*			*TaSUT2D*				
E value[b]	Identity (%)	Match length (bp)	E value	Identity (%)	Match length (bp)	E value	Identity (%)	Match length (bp)	Best hit contig	Contig's chromosomal location
0	100	739	0	100	739	0	99	741	#2683050	5D
0	97	739	0	97	739	0	97	741	#2242458	5B
0	92	526	0	92	526	0	93	526	#2270208	5B
$2e^{-86}$	99	183	$2e^{-86}$	99	183	$2e^{-86}$	99	185	#1490385	5A
$3e^{-77}$	99	168	$3e^{-77}$	99	168	$3e^{-77}$	99	168	#1540422	5A
$3e^{-77}$	99	168	$3e^{-77}$	99	168	$3e^{-77}$	99	168	#1508214	5A
$4e^{-44}$	83	182	$4e^{-44}$	83	182	$2e^{-42}$	81	186	#2749442	5D

[a]The IWGSC survey sequence repository was searched for contiguous DNA sequences (contigs) with the cDNA sequences of *TaSUT2A*, *TaSUT2B* and *TaSUT2D* as queries. [b]E Value determines the significance of the alignment score.
