# Supplementary material for: Identification and characterization of the three homeologues of a new sucrose transporter in hexaploid wheat (Triticum aestivum L.)
Source: BMC Plant Biol. 2013 Nov 16;13:181. doi: 10.1186/1471-2229-13-181 (PMC4225610; doi:10.1186/1471-2229-13-181)

Figure S1. Amplification of DNA fragments unique to each homeologue of TaSUT2. Polyacrylamide gel separation of the three distinct DNA fragments produced from tissues of cv. AC Andrew by a primer set designed to span polymorphic 3′ UTR region of the three homeologues. Lanes 1, 2 and 3 shows PCR products of expected sizes derived from each homeologue, and lane 4 represents the DNA ladder.

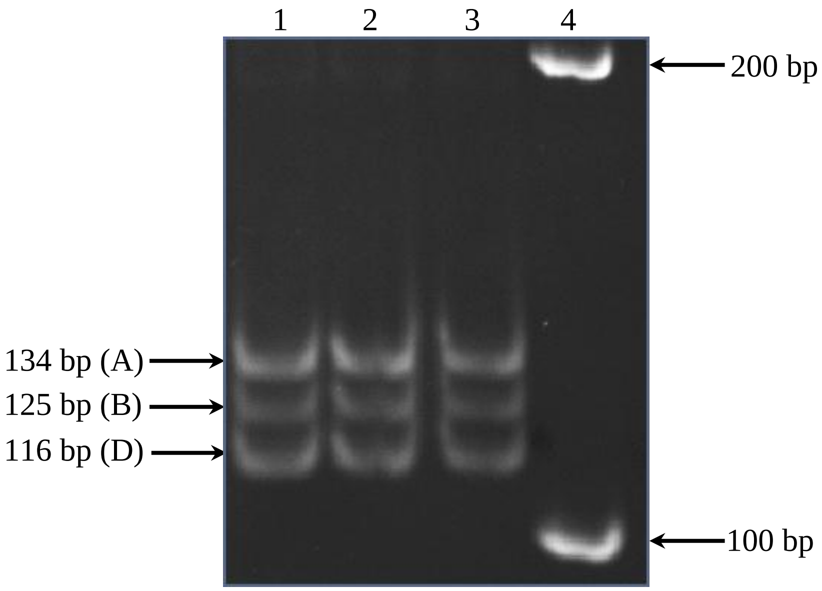

Supplement: Additional file 2: Figure S1 — Amplification of DNA fragments unique to each homeologue of TaSUT2. Polyacrylamide gel separation of the three distinct DNA fragments produced from tissues of cv. AC Andrew by a primer set designed to span polymorphic 3′ UTR regions of the three homeologues. Lanes 1, 2 and 3 shows PCR products corresponding to the amplicons of each homeologue, and lane 4 represents the DNA ladder. [file 1471-2229-13-181-S2.pdf]
